# Human Papillomavirus-Related Recurrent Multiphenotypic Sinonasal Carcinoma With HPV Genotype 56 Detected by HPV Direct Flow CHIP

**DOI:** 10.7759/cureus.40413

**Published:** 2023-06-14

**Authors:** Ana Lilia Remirez-Castellanos, Patricia Piña-Sanchez, Alejandra Mantilla-Morales, Wilfredo Valenzuela-Gonzalez, Fernando Candanedo González

**Affiliations:** 1 Radiology, Unidad Medico de Alta Especialidad (UMAE) Hospital de Oncologia, Centro Medico Nacional Siglo XXI. IMSS, Mexico, MEX; 2 Medical Research Unit in Oncological Diseases, Unidad Medico de Alta Especialidad (UMAE) Hospital de Oncologia Centro Medico Nacional Siglo XXI. IMSS, Mexico, MEX; 3 Pathology, Unidad Medico de Alta Especialidad (UMAE) Hospital de Oncologia Centro Medico Nacional Siglo XXI. IMSS, Mexico City, MEX; 4 Medicine, Universidad Autonoma de Sinaloa, Sinaloa, MEX; 5 Pathology, Instituto Nacional de Ciencias Medicas y Nutricion Salvador Zubiran, Ciudad de Mexico, MEX; 6 Pathology, Unidad Medico de Alta Especialidad (UMAE) Hospital de Oncologia, Centro Medico Nacional Siglo XXI IMSS, Ciudad de México, MEX

**Keywords:** hpv direct flow chip, adenoid cystic carcinoma, recurrent disease, hpv genotype 56, multifenotypic sinonasal carcinoma

## Abstract

Human Papillomavirus-related multiphenotypic sinonasal carcinoma is a rare, and recently described neoplasm, defined by its association with high-risk Human Papillomavirus, which exclusively affects the sinonasal tract and simulates salivary gland tumors. Due to the infrequency of this neoplasm and the lack of knowledge of its pathological characteristics, it is susceptible to diagnostic error. We describe the clinical-radiological findings of a 54-year-old man with multiphenotypic sinonasal carcinoma related to Human Papillomavirus genotype 56. The diagnosis of multiphenotypic sinonasal carcinoma was suspected by light microscopy and was corroborated by immunohistochemistry and polymerase chain reaction (PCR) analysis. The patient was subsequently treated with 63.6 gray radiotherapies. He is currently alive after a follow-up of 20 months, with a recurrence of the disease. In conclusion, multiphenotypic sinonasal carcinoma is an unusual neoplasm, which is not well recognized and can be confused with adenoid cystic carcinoma. However, multiphenotypic sinonasal carcinoma should be included in the differential diagnosis as we encounter sinonasal tumors, which by histology present tubular, cribriform, and solid growth patterns, accompanied by dysplasia or carcinoma in situ in the superficial mucosa. In this case, it is necessary to perform immunohistochemistry for p16INK4A or PCR to confirm the presence of high-risk Human Papilloma Virus, which would confirm the diagnosis.

## Introduction

Human Papillomavirus (HPV)-related multiphenotypic sinonasal carcinoma (HMSC) was described in 2012 by Bishop et al. [1], known initially as carcinoma with similar characteristics to adenoid cystic carcinoma (ACC) related to HPV. Since 2017, it is considered an emerging entity in the fourth edition of the classification of head and neck tumors by the World Health Organization (WHO) [2,3]. It is a rare malignant neoplasm defined by its association with high-risk HPV, particularly with 16, 31, 33, 35, and 56, which exclusively affects the sinonasal tract and simulates ACC [2-4]. Histologically, it is a heterogeneous neoplasm, which is characterized by basaloid cells that are arranged in solid, lobular, cribriform patterns with microcyst formation and a focal tubular component. The superficial mucosa epithelium frequently has the stratification of the epithelium with accentuated atypia with changes resembling squamous dysplasia [1,5,6]. It is characterized by high-grade histology with destructive local growth, advanced stage with frequent recurrences [6,7]. However, due to the limited number of published cases, its biological behavior is not yet fully defined. Only three instances of lung metastases and finger metastases have been published previously in the English literature [8]. This is the first case in Mexico that describes the presence of brain metastases. Therefore, our goal is to describe the clinical-radiological findings of this entity in a 54-year-old man with HMSC genotype 56, with intracranial metastases and recurrence during its clinical follow-up.

## Case presentation

A 54-year-old man with type 2 diabetes mellitus associated with chronic kidney disease. Positive smoking of 36 years, up to six cigarettes a day. He began his current condition in 2015, with rhinolalia attributed to sinusitis with left nasal obstruction, associated with increased volume in the left nasal pyramid, without alterations in vision or eye movements. In February 2018, episodes of epistaxis, anosmia, and headache of frontal predominance were added, so he sought medical attention. On physical examination, an increase in volume was observed in the left nasal pyramid with obstruction and epiphora. A transnasal biopsy was performed with a histopathological report of tubular and cribriform ulcerated ACC grade 2.

**Figure 1:**
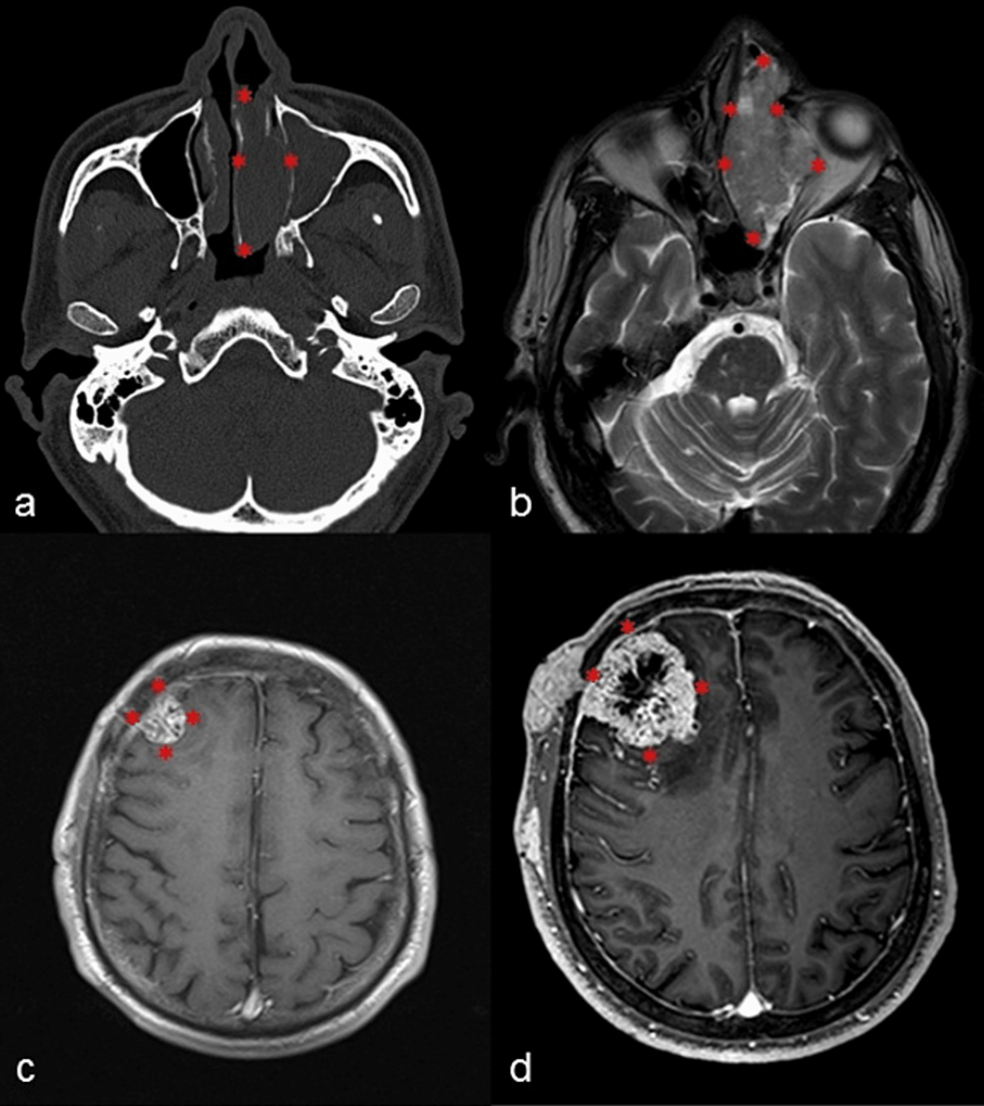
Images of the HMSC a) CT scan of a single axial skull with tumor lesion (asterisk) in an expansive hypodense nasal cavity with remodeling of the medial wall of the maxillary antrum, conditioning obstruction of the maxillary sinus, anterior and posterior ethmoid cells; b) T2 MRI amorphous tumor lesion in nasal cavity (asterisk) with extension to anterior and posterior ethmoid cells with infiltration to the medial wall of the orbit  c) Axial T1 MRI with contrast with extraaxial heterogeneous intracranial metastatic lesion (asterisk) with extension to meninges and soft tissues; d) T1 MRI with axial contrast five months later with disease progression (asterisk).

In March 2018, he was referred to the Centro Medico La Raza, where computed tomography (CT) and magnetic resonance image (MRI) were performed, which showed an amorphous tumor lesion affecting the left nasal cavity, with infiltration into the anterior and posterior ethmoid cells with cephalic expansion with the destruction of ethmoidal bone. The tumor extends medially to the nasal cavity and contralateral to the left orbit by displacing the medial rectus muscle without infiltrating it, expanding to the maxillary sinus, without infiltrating the adjacent cerebral parenchyma. In weighted sequences in T1, it is heterogeneously hypointense and hyperintense in T2, after administration of the contrast medium it presents intense reinforcement, with dimensions of 90x48x26 mm in the anteroposterior, cephalocaudal, and laterolateral axis (Figure 1). On April 25, 2018, a biopsy of the tumor in the left nostril was performed by the department of otorhinolaryngology, with an initial histopathological diagnosis of ACC with a solid, tubular, and cribriform pattern grade 2, therefore, he was referred to the Unidad Medico de Alta Especialidad (UMAE) Hospital de Oncologia Centro Medico Nacional Siglo XXI, to continue his management. In May 2018, tumor resection was performed through souther line incision: lateral rhinotomy incision (SOUTAR) type and Braxon RAY maneuver.

**Figure 2:**
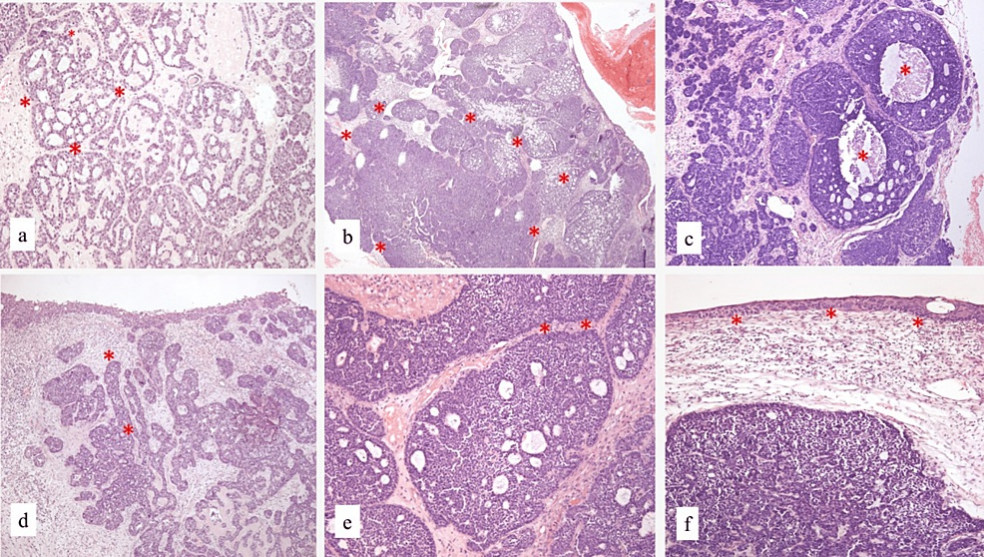
Morphology of the HMSC a) Heterogeneous neoplasm with cribriform areas (H&E; 100x; asterisk); b) mixed with solid areas (H&E; 100x; asterisk); c) Node with central necrosis type comedo (H&E;400x; asterisk); d-e) Cords and solid nests of basaloid cells (H&E; 100x; asterisk); f) Superficial epithelium with high grade dysplasia (H&E; 400x; asterisk).

The tumor was received multi-fragmented, which together measured 14.0x9.7x2.0 cm, was brown-gray, and had firm consistency. Partially covered by yellow-light mucosa. Microscopically, a solid and cribriform basaloid cell carcinoma with micro cysts was observed. Focally, comedo central necrosis was observed. Neoplastic cells showed round, uniform, and intermediate size nuclei with scarce cytoplasm. The overlying mucosa showed high-grade squamous dysplasia. Neither lymphovascular nor perineural invasion was identified (Figure 2).

**Figure 3:**
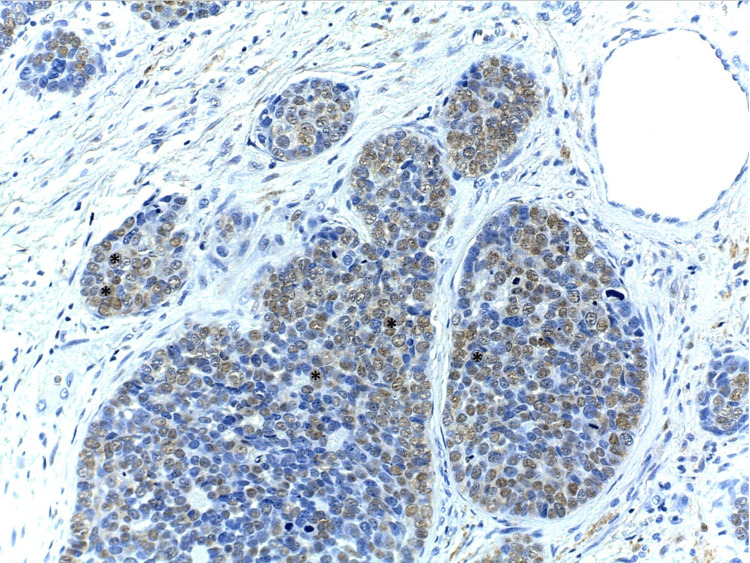
Immunohistochemistry with nuclear expression of p16 in neoplastic cells (H&E; 400x; asterisk).

Immunohistochemistry was performed for p16, as a surrogate marker for the presence of HPV. If staining was both nuclear and cytoplasmic and present in ≥70% of the tumor cells p16 immunostaining was regarded as positive. Neoplastic cells showed a positive reaction for p16INK4A with intense nuclear and cytoplasmic reactions (Figure 3).

HPV detection was performed using the HPV direct flow chip master diagnostic (IVD) technique. The technique is based on viral DNA amplification by polymerase chain reaction (PCR) and subsequent membrane hybridization by reverse dot-blot. This technique identifies 36 genotypes using the HybriSpot24 automated system (Vitro Group) [9]. In our case, HPV 56 was identified.

After the diagnosis of HMSC related to HPV genotype 56, neoadjuvant radiotherapy was added, 63.6 Grays in 30 fractions, with neck extension presenting cervical abscess as a complication. Currently, after 20 months of follow-up, the patient is asymptomatic. However, in the last MRI image study, tumor recurrence of irregular morphology was observed, with intermediate signal intensity in T1, higher signal intensity in T2, and fluid-attenuated inversion recovery (FLAIR) sequences, with avid enhancement after administration of the contrast medium. The tumor extends into the sphenoid sinus, left orbit contacting the medial rectus and superior oblique rectus muscle. Several extraaxial, supratentorial tumors of heterogeneous irregular morphology hypointense in T1 and hyperintense in T2 were identified, after administration of the contrast medium that showed heterogeneous enhancement of peripheral predominance with central necrosis. The largest lesion was found in the right frontal region, with another lesion of the same characteristics with bone involvement and ipsilateral meningeal reinforcement.

## Discussion

Sinonasal malignant neoplasms comprise only 3% of all malignant neoplasms of the head and neck [1,5,10]. Squamous cell carcinomas are the most common subtype [10]. On the other hand, there are several subtypes of sinonasal carcinomas related to HPV that are well recognized. Among those found is HMSC, originally known as carcinoma with characteristics similar to HPV-related ACC [1,2]. In the original series of HPV-related ACC, Bishop et al. [1]., described eight cases. Actually, only 56 cases have been reported in English literature [1,4,11-14]. This makes it a rare neoplasm that due to its low frequency is little known. In most reports, it most frequently affects the female gender with an average age of 54 years (range, 28-90 years) [1,5]. Approximately 20-25% of sinonasal carcinomas are associated with high-risk genotypes, specifically HPV genotype 33. Most of the cases presented are in advanced clinical stages and almost all have a high-grade histological aspect [15]. About 90% of cases originate in the sinonasal tract associated with a clinical presentation of nasal obstruction and epistaxis. As in our case it manifests with obstructive symptoms of three years of evolution.

The term multiphenotypic describes a heterogeneous neoplasm with differentiation towards multiple cell lineages [1,5]. One of the characteristics of HMSC is the presence of basaloid myoepithelial cells that are arranged in solid mantles, lobes, and cribriform-looking nests [6]. Many of the HMSCs show high-grade characteristics, with a high mitotic rate and tumor necrosis [1,5]. In our case, we observed a high mitotic index, in the absence of tumor necrosis. Another characteristic is that they can show squamous differentiation with squamous dysplasia of the superficial epithelium that covers the sinonasal tract and less frequently, as an infiltrating component with keratin production [1,5]. Therefore, in the sinonasal region, it is important to consider among the differential diagnoses ACC, basaloid squamous carcinoma, high-grade myoepithelial carcinoma, SMARCB-1 (INI-I) deficient sinonasal carcinoma, and NUT midline carcinoma V [14].

In our case, it was initially confused with ACC grade 2, both in the first review of biopsy and in the case reassessment, since both the solid and the cribriform pattern can be confused with the solid variant of ACC [1,5,16]. Only about 10 to 25% of all ACC originated in the sinonasal region (SNACC). SNACCs manifest as an indolent mass of slow growth, they are associated with frequent local recurrences and distant metastases. Therefore, they present a worse outcome, with a five-year survival rate of 62%. They are characterized by a myoepithelial and ductal biphasic differentiation with tubular, cribriform, and solid architecture. Myoepithelial cells have dark-angled nuclei with scarce cytoplasm, which is a basaloid aspect. The cribriform pattern is predominantly composed of myoepithelial cells with hyalinized or myxoid cells. The solid pattern presents solid nests composed of a sheet of basaloid cells. In a few cases, high-grade transformation can be observed, with comedo-like tumoral necrosis, more than 10 mitoses in 10 high-power fields with marked nuclear atypia. Occasionally there can be focal squamous metaplasia that associates with a higher risk for lymphatic node metastases, distant metastases, and disease-related death. It was in the extensive sampling of tumor resection and confirming HPV expression in the neoplasm that it was demonstrated that it was an HMSC. When compared, ACC most often shows invasion and metastasis (up to 40 to 50% of cases) [14]. While in the HMSC variant, perineural infiltration is uncommon [17]. Thus, the results of the different cases have shown that HMSC has a better prognosis than ACC [15].

The immunohistochemical method does not allow the separation of HMSC from adenoid cystic carcinoma because both tumors share the presence of myoepithelial cells that express calponin, p63, p40, actin and S100 protein and ductal cells that express c-kit [6]. However, another characteristic of multiphenotypic sinonasal carcinoma is its association with HPV infection, with high-risk genotypes 16, 31, 33, 35, and 56 [1,4,11-14]. Also by IHC, p16INK4A has been used as an HPV subrogate with a strong and diffuse, nuclear and/or cytoplasmic expression in more than 70% of the neoplastic cells, unlike the ACC that is negative [1]. In our case, we found a positive reaction to p16INK4A with an intense nuclear and cytoplasmic reaction. Other more rigorous detection methods for the detection and genotyping of HPV in multiphenotypic sinonasal carcinomas are PCR and hybridization assays. These trials have shown that multiphenotypic sinonasal carcinomas are associated with HPV genotypes 16, 33, 35, and 52 [1,4,11-14]. It is noteworthy that genotype 16, which is usually the most frequent in oropharyngeal epidermoid carcinomas that are HPV positive, is not the most frequent in HMSC. In most cases that have been reported, HPV genotype 33 has been identified [6].

Regarding the biological behavior of HMSC, it is not yet well characterized. In the initial publication of Bishop et al. [1]., they analyzed eight cases of HMSC. They observed that all tumors were located in the primary site without evidence of local or distant metastases. At the time of presentation, the patients were in stages T1N0M0 (n = 3), T3N0M0 (n = 1), and T4N0M0 (n = 3) [1]. Subsequently, in a second study gathered forty-nine patients of which 41% of the cases were in stage T1, 17% in stage T2, 21% in stage T3, and 23% in T4 (5). No evidence in any of the cases of local or remote dissemination. The duration of follow-up varied from one to 256 months (mean, 42 months). Fourteen of 39 (36%) patients developed local recurrences. Recurrences occurred 23 to 130 months after the original treatment. Two of 39 (5%) patients developed distant metastases at 96, 144, 192, and 204 months after treatment. The metastatic sites involved were the lung in two patients and the finger in one patient. At the last known follow-up, 30 of 49 patients were alive without evidence of disease and nine patients were alive with disease. None of the patients developed lymph node metastases or died as a result of their disease [5]. In our case, after 18 months of follow-up, the patient developed a recurrence of loco-regional disease twice. Currently presents intracranial metastases and leptomeninges, with involvement in the surgical bed, which suggests the possibility of tumor sowing secondary to the surgical approach. However, we consider that in order to better understand their biological behavior and due to their rarity, it is necessary to carry out a multi-institutional study that gathers a greater number of cases that allows a more reliable study of their clinical-pathological characteristics in the Mexican population. Another interesting aspect to evaluate is smoking exposure; It has been reported in patients with squamous cell carcinoma of the head and neck, that cases positive for HPV, associated with smoking, have a negative impact on local and distant recurrence [18].

Through CT, and MRI studies of the HMSC, they appear to be indistinguishable from conventional sinonasal epidermoid carcinomas. However, of the 56 cases reported in the English literature, only four cases of HMSC reported with histopathological confirmation and molecular test, imaging findings have been described [3,11-14]. In all cases, it has been observed that they are tumors that by the image are invading bone, destroying and remodeling. By RM, they have a variable signal in relation to the cortex in T2WI. The radiological differentiation of the sinonasal malignant tumors is difficult due to the similarity of the imaging findings, the location of the tumor, the growth pattern in the adjacent bone, tumor homogeneity, signal intensity, contrast enhancement, diffusion weighted imaging (DWI), and apparent diffusion coefficient (ADC) can facilitate the right diagnosis. CT and risk of malignancy index (RMI) are useful tools for the evaluation of the previous treatment of the characterization, and location of these entities [17].

Table 1 summarizes the clinical-pathological characteristics of all reported cases of multiphenotypic sinonasal carcinoma related to HPV.

**Table 1:** Summary of the clinical-pathological characteristics of all reported cases of multiphenotypic sinonasal carcinoma related to HPV. Gender: M - Male, F- Female; CT: computed tomography; MRI: magnetic resonance image; T: Tumor, N: Nodes, M: Metastases.

Reference	# cases	Age	Gender	Genotype	Clinical Stage	Image	Size	Time of evolution (months)	Recurrence	Survival (months)
Bishop et al. 2013 [1]	8	40-73	6F	31-33 (n=5)	T1N0M0 (n=3)	No	1.1-5.4	No	Local (n=2)	15
											2M	16 (n=2)	T3N0M0 (n=1)
			T4N0M0 (n=3)									
			Brzezinska et al. 2019 [3]	1	70						1M	High risk	T3N0M0	MR: Expansive heterogeneous tumor lesion in nasal cavity.	7.8	11	No	2
Bishop et al. 2017 [5]	49	28-90				25F	56 (n=1) 16 (n=2)	T1 N0M0 (n=16) T2N0M0 (n=7) T3N0M0 (n=8) T4N0M0 (n=9)	No	0.7-8.5									No	Local (n=14) Distal (n=2) Lung Finger	42
			21M	33 (n=1) 35 (n=3)																	
					Ruanggritchankul et al. 2018 [10]						1	50	1F	26 (n=1)	T2aN0M0	No	No	6				No	85
						Hwang et al. 2015 [11]	1																	75	1F	33	T1N0M0	CT: Tumor lesion on the inferior turbinate. MR: Isointense lesion in T1 and heterogeneous T2.	No.	3	No	12
			Chouake et al. 2018 [12]	2	60						1M	35 (n=1)	No	No	3.5	No	No	No														
					46						1M	33 (n=1)																				
Adame et al. 2019 [13]	1	48	1F	52 (n=1)	T1N0M0	MRI: Expansive tumor lesion in the left nasal cavity insointense in T2.	3.5	6	No	12
											Ching et al. 2019 [14]	1	54	1F	16 (n=1)	No	CT: Expansive homogeneous tumor lesion, bone remodeling.	No.	3	No	9
Remirez et al. 2019 [Present case]	1	52	1M	56 (n=1)	T3N0M0	MRI: Tumor injury amorphous infiltrant hypointense in T2 hyperintense in T2.	3	36	Local and intracraneal and Meninges.	18											

## Conclusions

HMSC is an unusual neoplasm, which is not well recognized and can be confused with adenoid cystic carcinoma. However, multiphenotypic sinonasal carcinoma should be included in the differential diagnosis as we encounter sinonasal tumors, which by histology present tubular, cribriform, and solid growth patterns, accompanied by dysplasia or carcinoma in situ in the superficial mucosa. In this case, it is necessary to perform immunohistochemistry for p16INK4A or PCR to confirm the presence of high-risk Human Papilloma Virus, which would confirm the diagnosis. In rare occasions, it can present a more aggressive biological behavior with recurrence and cerebral metastases, as it was in our case. 
